# Reliability of Task-Based fMRI for Preoperative Planning: A Test-Retest Study in Brain Tumor Patients and Healthy Controls

**DOI:** 10.1371/journal.pone.0149547

**Published:** 2016-02-19

**Authors:** Melanie A. Morrison, Nathan W. Churchill, Michael D. Cusimano, Tom A. Schweizer, Sunit Das, Simon J. Graham

**Affiliations:** 1 Physical Sciences Platform, Sunnybrook Research Institute, Toronto, ON, Canada; 2 Department of Medical Biophysics, University of Toronto, Toronto, ON, Canada; 3 Keenan Research Centre, St. Michael's Hospital, Toronto, ON, Canada; 4 Division of Neurosurgery, St. Michael's Hospital, Toronto, ON, Canada; 5 Department of Surgery, University of Toronto, Toronto, ON, Canada; University of Texas at Austin, UNITED STATES

## Abstract

**Background:**

Functional magnetic resonance imaging (fMRI) continues to develop as a clinical tool for patients with brain cancer, offering data that may directly influence surgical decisions. Unfortunately, routine integration of preoperative fMRI has been limited by concerns about reliability. Many pertinent studies have been undertaken involving healthy controls, but work involving brain tumor patients has been limited. To develop fMRI fully as a clinical tool, it will be critical to examine these reliability issues among patients with brain tumors. The present work is the first to extensively characterize differences in activation map quality between brain tumor patients and healthy controls, including the effects of tumor grade and the chosen behavioral testing paradigm on reliability outcomes.

**Method:**

Test-retest data were collected for a group of low-grade (n = 6) and high-grade glioma (n = 6) patients, and for matched healthy controls (n = 12), who performed motor and language tasks during a single fMRI session. Reliability was characterized by the spatial overlap and displacement of brain activity clusters, BOLD signal stability, and the laterality index. Significance testing was performed to assess differences in reliability between the patients and controls, and low-grade and high-grade patients; as well as between different fMRI testing paradigms.

**Results:**

There were few significant differences in fMRI reliability measures between patients and controls. Reliability was significantly lower when comparing high-grade tumor patients to controls, or to low-grade tumor patients. The motor task produced more reliable activation patterns than the language tasks, as did the rhyming task in comparison to the phonemic fluency task.

**Conclusion:**

In low-grade glioma patients, fMRI data are as reliable as healthy control subjects. For high-grade glioma patients, further investigation is required to determine the underlying causes of reduced reliability. To maximize reliability outcomes, testing paradigms should be carefully selected to generate robust activation patterns.

## Introduction

Over the last two decades, functional magnetic resonance imaging (fMRI) has evolved as a powerful neuroimaging technique, providing detailed maps of brain activity derived from hemodynamic responses to neuronal activity. Although fMRI is used primarily as a neuroscience research tool, clinical applications are emerging, with the most mature involving the use of fMRI as a preoperative planning tool for brain tumor and epilepsy surgery [1–3]. The brain activation maps obtained by fMRI can help the surgeon to identify regions involved in motor control [4] and language function [5], including high-risk areas that, if damaged during surgery, would likely result in a significant neurological deficit. Furthermore, such fMRI data may directly influence the surgical management of patients in many possible ways, such as to help determine the most feasible treatment option (e.g. craniotomy performed with or without intraoperative mapping); the necessary extent of brain exposure; the safest surgical entry point and corridor; the selection of intraoperative tasks during direct cortical stimulation (DCS); and the need for sub-cortical DCS [4,6–8].

Despite these early promising results, there are still concerns about whether fMRI in patients with brain tumors is sufficiently reliable for clinical applications. Brain activation maps may be impacted by the choice of experimental design, the magnitude of the blood oxygenation level-dependent (BOLD) response, confounding noise (e.g. head motion), image processing pipelines and statistical analysis methods [8,9]. Clinical imaging is further complicated by the effects of tumor on the BOLD signal, and by increased head motion often exhibited in patient populations in comparison to healthy controls [10].

The issue of fMRI reliability in normal subjects (i.e. the level of reproducibility measured within fMRI data acquired from multiple test runs) has been studied extensively by the experimental neuroimaging community. Many studies have been undertaken involving healthy cohorts using test-retest methods [11], although to date, very little data have been obtained directly in patients with brain tumors. Furthermore, the majority of fMRI test-retest studies quantify reliability based on the spatial overlap of brain activation using the Dice and Jaccard coefficients, and the consistency of global signal effects using the intra-class correlation coefficient [12–14]. Despite the robustness and entrenched use of these metrics, taken alone they do not provide a comprehensive evaluation of fMRI reliability. Characterizing reliability using other metrics is likely to be important for the specific case of preoperative fMRI applications. Candidate metrics include the center of mass (COM) of pertinent brain activation clusters, the laterality index, and the lesion-to-activation distance (LAD). In particular, the latter metric can only be assessed properly in the brain tumor patient population. Furthermore, the role of preoperative fMRI in surgical decision-making needs to be carefully considered as part of the reliability assessment.

To address the shortcomings of previous test-retest studies in relation to preoperative fMRI in brain tumor patients, our work reports on a cohort of brain tumor patients and patient-matched healthy controls who repeated a battery of motor and language tasks during a single fMRI session. Test-retest reliability was investigated by applying a novel single-subject preprocessing pipeline optimization algorithm that yields state-of-the-art activation maps [15,16] and using a variety of metrics to make inferences about reproducibility within and across cohorts (i.e. low-grade vs. high-grade tumors, patients vs. controls), as well as across different testing paradigms (i.e. motor task vs. language task, language task A vs. language task B).

## Materials and Methods

### Subjects

With approval from the Research Ethics Boards at Sunnybrook Health Sciences Centre, Toronto, Canada, and St. Michael’s Hospital, Toronto, Canada, eighteen brain tumor patients (10 male, 8 female; mean age 43.2 ± 13.7) were recruited to participate in this research study. Recruitment criteria included: clinical or radiological evidence of probable low-grade glioma (LGG; World Health Organization (WHO) grades I-II) or high-grade glioma (HGG; WHO grades III-IV) near or within eloquent brain regions (i.e. sensory, motor, or language areas), no contraindications to MRI (e.g. severe claustrophobia, metallic implants), and no other major neurological or psychological disorder. Patient demographics are listed in Table 1.

**Table 1:** Patient Demographics (M = male, F = female, L = left, R = right). Brain tumor anatomy for each patient is shown in Fig 1.

Patient No.	Age/Sex/Handedness	Tumor Grade/ Pathology	Tumor Location	Preoperative language or motor deficit?
P01	53/F/R	IV/Glioblastoma	R-temporo-parietal	Moderate speech deficit
P02	55/M/R	IV/Glioblastoma	R-temporo-parietal	Mild speech deficit
P03	22/M/R	IV/Glioblastoma	R-parietal	Moderate word processing deficit
P04	57/F/R	IV/Glioblastoma	L-temporal	Severe speech and motor deficit (bilateral leg weakness)
P05	43/M/L	IV/Glioblastoma	L-parietal	No
P06	25/F/L	III/Anaplastic Astrocytoma	R-frontal	No
P07	48/F/R	III/Gemistocytic Astrocytoma	L-fronto-insular	Moderate speech deficit
P08	60/M/R	II/Oligodendroglioma	L-frontal	No
P09	36/M/R	II/Astrocytoma	R-frontal	No
P10	40/M/L	IV/Glioblastoma	R-frontal	No
P11	54/M/R	Metastatic Adenocarcinoma (Lung)	L, R-parietal	Moderate motor deficit (right leg twitch)
P12	47/F/R	II/Low-grade glioma	L-frontal	No
P13	27/M/R	II/Oligodendroglioma	R-frontal	No
P14	38/F/R	II/Oligodendroglioma	R-frontal	No
P15	23/F/R	I/Ganglioglioma	L-insular	No
P16	45/M/R	II/Low grade glioma	R-frontal	No
P17	35/F/R	II/Astrocytoma	L-parietal	No
P18	70/M/R	IV/Glioblastoma	R-temporo-parietal	Mild speech deficit

**Fig 1:**
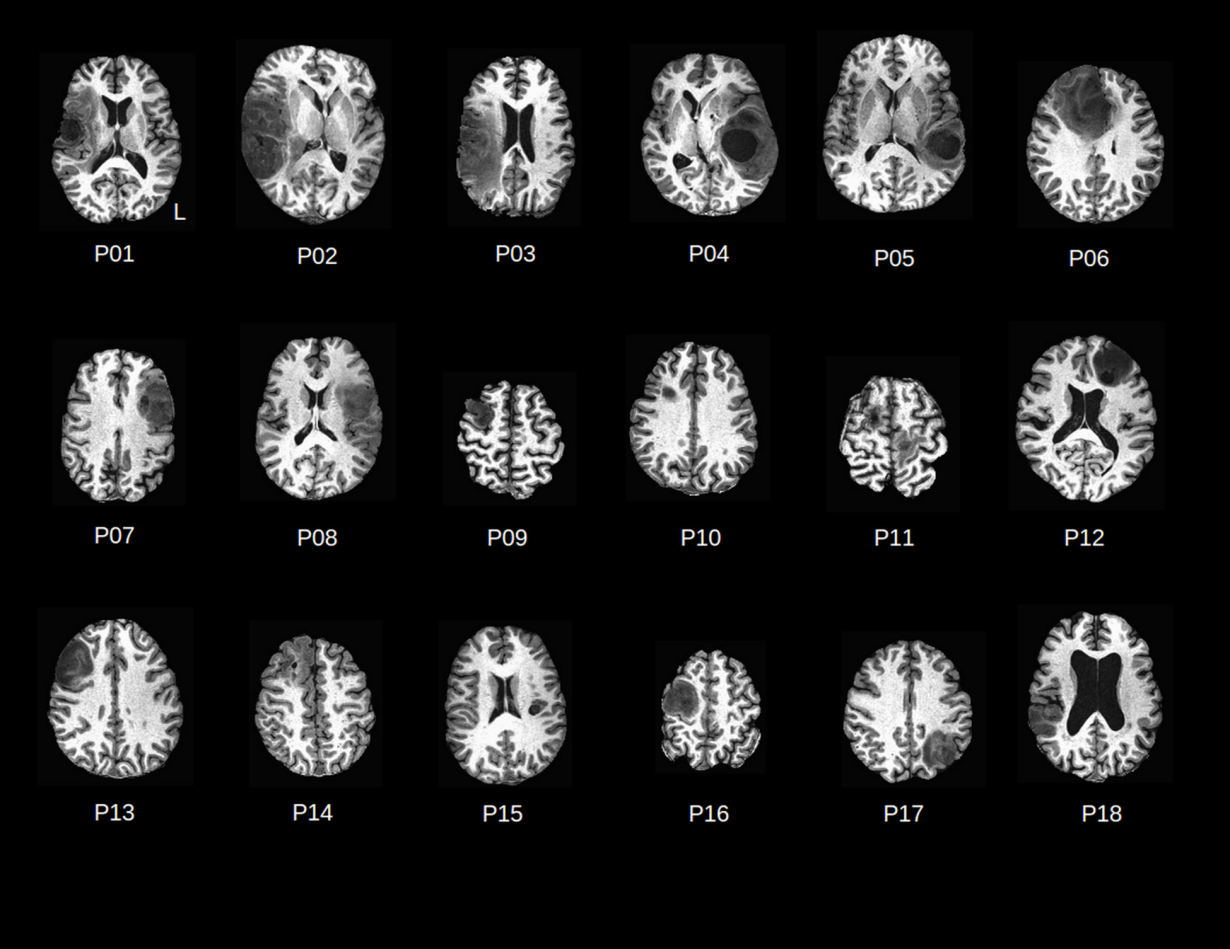
T1-weighted axial MRI slices displaying patient brain tumor anatomy (P01-P18).

All but three patients were right-handed. On histopathology of subsequent surgical samples, 9 patients were found to have HGG, 8 to have LGG, and one patient to have brain metastases from primary lung cancer. Apart from this patient (P11) who presented with left and right brain metastases, there were 10 right hemispheric tumors and 7 left hemispheric tumors (Fig 1). For each patient, one healthy control matched in age (± 2 years), sex, and handedness was also recruited to the study.

For each volunteer subject, fMRI was performed during a single visit to Sunnybrook Research Institute (SRI), Toronto, Canada. Prior to participation, informed written consent was obtained from each subject and a 15-minute training session was undertaken to allow subjects to familiarize themselves with the behavioral tests to be performed, as well as with an fMRI-compatible tablet system that was used for test delivery and subject performance [17]. The tablet technology was equipped with a writing stylus and computer software (E-Prime, Psychology Software Tools, Sharpsburg, PA) capable of administering a variety of cognitive tasks. Lying in the magnet bore, the tablet platform rested over the torso at a comfortable angle for interaction. Visual stimuli were transmitted to the subjects via a liquid crystal display projector (Avotec, Inc., Stuart, FL) onto a rear-projection screen that was visible through an angled mirror attached to the head coil (diagonal viewing angle, 25.3°). The tablet system was used for this test-retest fMRI study because the technology has recently been implemented into awake craniotomy procedures to expand the behavioral testing repertoire available during intraoperative mapping, and to improve standardization of behavioral tests during pre- and intra-operative mapping [18].

### Image Acquisition

All subjects were imaged using a research-dedicated 3T MRI system (MR750, GE Healthcare, Waukesha, WI) equipped with an 8-channel head receiver coil and peripherals for recording cardiac and respiratory signals (photoplethysmograph and bellows, respectively). The protocol consisted of initial localizer images followed by IR-FSPGR (inversion recovery prepared fast spoiled gradient echo) T1-weighted axial anatomical imaging (repetition time (TR)/echo time (TE)/flip angle (Ɵ)) = 82 ms/3.2 ms/8 degrees); field of view (FOV) = 22 cm × 22 cm; 190 slices; slice thickness = 1 mm) and fMRI using repeated spiral in/out T2*-weighted imaging (TR/TE/Ɵ = 2000 ms/30 ms/70 degrees; FOV = 20 cm × 20 cm; 30 slices; slice thickness = 4.5 mm) to record BOLD hemodynamic responses to neural activity effect [19]. During the 1-hour imaging session, patients performed a battery of up to three behavioral tests in duplicate, with a 20 minute test-retest interval time; identical procedures were used for the matched healthy controls. The randomized battery consisted of a hand motor task (“hand squeezing”), a rhyming task [20] and a written phonemic fluency task [21], as described below.

### fMRI Task Battery

#### Hand Squeezing (HS)

Subjects were given a latex squeeze toy in the hand contralateral to the hemisphere of tumor dominance (control subjects used the same hand as their patient match) and instructed to squeeze continuously at a self-directed, comfortable pace. Eight 15 s task blocks alternated with 15 s blocks of rest for a total run time of 240 s. The toy generated a ‘squeak’ sound that was audible from the MRI console, providing an appropriate monitor of task performance.

#### Rhyming Words (RW)

Subjects were presented with a pair of words and instructed to decide silently if the word pairs rhymed. Forced-choice “yes” or “no” responses were recorded by pressing an icon on the tablet, and monitored from a computer at the MRI console. During each 18 s task block, 6 different word pairs were each displayed for 3 s. Eight separate task blocks were alternated with a baseline condition, in which line pairs were shown and subjects had to determine if the line pairs were alike in volume and orientation. The total run time was 300 s. The procedure for making tablet responses during the baseline condition and during the rhyming task was identical.

#### Phonemic Fluency (PF)

Subjects were presented with a single letter and given 60 s to produce as many words as possible beginning with that letter. Written responses were recorded on the tablet using the stylus, and monitored from the MRI console. Three PF task blocks were administered, alternating with a 20 s baseline condition that involved writing varying lengths (self-chosen) of symbol strings composed of double-loops (e.g. “8”, “88”, “888”, etc.), then followed by 16 s of rest. Different 3-letter combinations of equal task demands (C-F-L and P-R-W) [22] were used for the test and re-test run to minimize memory and learning effects.

### Data Analysis

Preprocessing was performed within the NPAIRS (Nonparametric, Prediction, Activation, Influence, and Reproducibility reSampling) framework [16]. For both patient and control groups, this provided optimized single-subject preprocessing pipelines that yielded the most reproducible and task-predictive activation maps. In this framework, the quality of fMRI data was evaluated for a given preprocessing pipeline by splitting the task run temporally into two halves and analyzing the split-halves independently. Reproducibility (R) was computed as the Pearson correlation between split-half activation maps; prediction (P) was computed by using the analysis model of one split-half to classify scans from the other split-half, based on Bayesian posterior probability. The (P,R) measures were computed for all possible combinations from a set of pipeline steps turned on/off (see immediately below), and the combination minimizing the Euclidean distance measure $\sqrt{{\left(1-P\right)}^{2}+{\left(1-R\right)}^{2}}$ was selected as the optimal pipeline, for each subject and task run [15]. The series of pipeline choices, some of which were extracted from the analysis of functional neuroimages (AFNI) software package (version: 2011_12_21_1014) [23], included: motion correction (AFNI, *3dvolreg*), outlier censoring and interpolation [24], physiological correction of cardiac and respiratory data (AFNI, *3dretroicor*), slice-timing correction (AFNI, *3dTshift*), spatial smoothing with an isotropic 6 mm Gaussian filter (AFNI, *3dmerge*), temporal detrending (0^th^-3^rd^ order Legendre polynomials), motion parameter regression (using Principal Components of the 6 rigid-body motion parameters that account for >85% of motion variance), task covariates, global signal removal by regressing the first Principal Component of the fMRI data [25], and data-driven physiological correction using the PHYCAA+ algorithm [26].

For all tested processing pipelines, a univariate Gaussian Naïve Bayes model was used to estimate brain activity; this is the predictive formulation of the widely-used General Linear Model (GLM), which allows us to measure prediction accuracy of pipelines. Correction for multiple comparisons was performed using the false discovery rate [27] threshold of q < 0.05 and applied to the whole brain and region-of-interest (ROI) activation maps. The maps for individual subjects were then scrutinized with a small variable threshold, t_s_, (z-score < 1) to minimize any residual supra-threshold voxels potentially caused by artifact, and to stabilize active clusters and their spatial extent. Datasets were also created for two additional thresholds, t_s_- and t_s_+, at ± 20% of the initial threshold, such that reliability metrics could be evaluated at multiple thresholds. This approach has been previously used in the literature to good effect, as it minimizes errors in interpreting activation maps associated with a fixed threshold [28].

**Fig 2:**
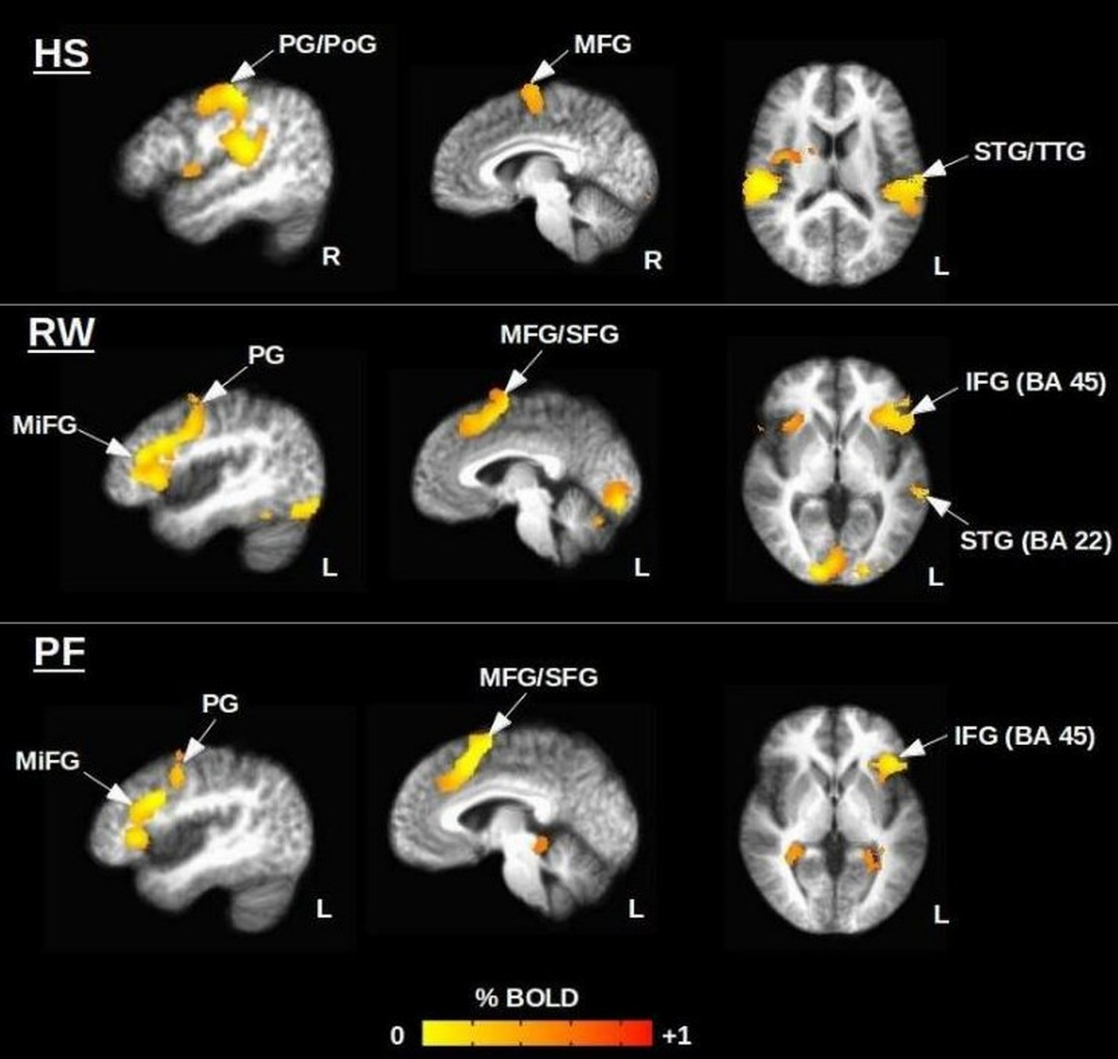
Healthy control group maps (n = 12) for the Hand Squeezing (HS), Rhyming Words (RW), and Phonemic Fluency (PF) tasks. Peak coordinates are provided in Table 2. Abbreviations: PG, PoG = precentral, postcentral gyrus; SFG, IFG, MiFG, MFG = superior, inferior, middle, medial frontal gyrus; STG, TTG = superior, transverse temporal gyrus. BA = Brodmann area.

The procedure for delineating each ROI started with visual assessment of the group average t-statistic data for controls, which showed the expected patterns of activity for each fMRI task (Fig 2, Table 2). Brain regions labelled in Fig 2 for each task (e.g. precentral gyrus, middle frontal gyrus) were identified from the Talairach-Tournoux Atlas (N27) and combined to create a mask of all ROIs. This was done using the “draw dataset” plugin built into the AFNI graphical user interface (GUI). The affine transformation of native spatial coordinates to Talairach coordinates, and *vice versa*, was fully automated and immediately updated on the GUI. When anatomical regions were shifted or distorted in patients by the tumor volume, ROIs were modified manually by one experienced individual highly knowledgeable in functional neuroanatomy (M.M).

**Table 2:** Peak Talairach coordinates of activated brain regions for the hand squeezing (HS), rhyming (RW), and phonemic fluency (PF) tasks. Abbreviations: SFG, IFG, MiFG, MFG = superior, inferior, middle, medial frontal gyrus; STG = superior temporal gyrus.

Talairach Coordinates (mm)	Brain Regions	Brodmann Area	Peak t-statistic		
			HS	RW	PF
**Left Hemisphere**					
(-48,-30,14)	STG	41	7.0		
(-42,-3,35)	Precentral gyrus	6		7.6	
(-44,18,22)	MiFG	9, 46		4.8	
(-2,5,53)	SFG	6		6.3	
(-45,20,4)	IFG	45 (Broca)		7.1	
(-55,-29,4)	STG	22 (Wernicke)		6.3	
(-43,-2,42)	Precentral gyrus	6			5.7
(-43,12,28)	MiFG	9, 46			5.9
(-3,11,47)	MFG	6			6.8
(-43,20,4)	IFG	45 (Broca)			5.8
**Right Hemisphere**					
(49,-13,44)	Precentral gyrus	4	7.8		
(7,-9,61)	MFG	6	6.4		
(52,-27,15)	STG	41	8.7		

### Metrics of Test-Retest Reliability

To assess test-retest reliability in the cohorts of brain tumor patients and matched healthy controls, a comprehensive set of metrics was adopted. The reliability of single-subject maps was characterized by the spatial overlap and displacement of brain activity clusters, the stability of the BOLD signal, and the laterality index across test runs, and re-test runs. In addition to exploring group differences in test-retest reliability between patients and controls, these metrics were also used to explore the difference in reliability between fMRI tasks (i.e. motor vs. language, and language vs. language). Lastly, the effect of tumor stage on fMRI reliability was assessed by comparing results for HGG patients (n = 6) and LGG patients (n = 6). Although such a comparison has not been reported in the literature to date, it was expected that HGG patients would show decreased fMRI reliability in relation to LGG patients, in keeping with the effects of more aggressive and invasive disease, and likely greater disruption of neurological function. For all comparisons, statistical testing was performed using a two-sided Wilcoxon rank sum test at the p<0.05 significance level. A description of the individual metrics is provided below.

#### Spatial overlap

The degree of spatial overlap of active voxels was measured between test and retest sessions via the Jaccard Index, J_o_, and the Dice coefficient, D_o_:

$$J_{o}=\left[\frac{V_{o}}{V_{1}+V_{2}-V_{o}}\right]x\;100,$$ [1]

$$D_{o}=\left[2\;x\frac{V_{o}}{V_{1}+V_{2}}\right]x\;100$$ [2]

where V_o_ is the number of overlapping voxels across test sessions, and V_1_ and V_2_ are the number of active voxels in the individual test sessions, respectively. These resulting values are often interpreted as the ‘percentage overlap’, ranging between 0% (no overlap) and 100% (perfect overlap). The J_o_ measure tends to estimate lower overlap than D_o_, with the greatest differences occurring at intermediate overlap values; the D_o_ measure is also known to be susceptible to “aliasing” effects, where different patterns produce highly similar overlap values [29]. Both J_o_ and D_o_ were calculated at the whole brain and ROI level, and the average across t_s_, t_s_-, and t_s_+ was computed to yield final values for each subject.

#### Cluster displacement

The displacement of brain activity clusters across test and retest runs was computed by a Euclidean distance measurement. At the primary threshold, t_s_, where clusters were identified as most stable (minimal changes in cluster extent and volume) for individual subjects, the 3dclust command in AFNI (first-nearest neighbour; cluster threshold = 5 voxels) was used to extract centre of mass (COM) and peak coordinates for clusters residing within the selected ROIs. Each cluster maintaining a unique spatial position in both the test and re-test activation maps had a COM and peak displacement measure recorded, which was then averaged with measures from other nearby clusters. Lower displacement values indicated better reliability for this measure.

#### Stability of BOLD signal

The stability of the BOLD signal amplitude was calculated based on the t_diff_ metric adopted by Gorgolewski *et al*. (2013) to evaluate the between-session variance of t values for single-subject test-retest data [30]. The metric is calculated simply from unthresholded t-statistic maps and is inversely related to calculation of the intra-class correlation coefficient (ICC) for 2 sessions. For each task, t_diff_ was computed across the whole brain and within selected ROIs according to

$$t_{diff}=\frac{1}{n}\sum_{i=1}^{n}{\left(t_{i1}{-t}_{i2}\right)}^{2}$$ [3]

where *n* is the number of voxels and t_i1_ and t_i2_ are the i^th^ voxel t-values of brain activity in the first and second test sessions, respectively. Test-retest data with lower t_diff_ values are interpreted as more reliable, whereas higher t_diff_ values indicate less reliability.

#### Laterality index

Test-retest reliability was also assessed with respect to within-subject laterality of brain activity across the two cerebral hemispheres, given the usefulness of laterality assessments in preoperative fMRI. The assessment was made in terms of the laterality index (LI) according to

$$LI=\frac{N_{left}-N_{right}}{N_{left}+N_{right}}$$ [4]

where N_left_ and N_*right*_ represented brain map quantities from the left and right hemisphere, respectively. These brain map quantities are commonly characterized according to the extent of activated brain volume (number of active voxels in ROIs), LI_e_; or by brain map signal intensity (mean or sum of t values or β coefficients of active voxels in ROIs), LI_m_ [31]. It has been argued that the latter is a more robust measure of laterality, although Jansen *et al*. (2006) reported that reproducibility was comparable for LI_e_ and LI_m_ measured in healthy subjects. Given these findings, both LI_e_ and LI_m_ (based on the sum of t value) are reported in the present work to compare with previous reports while making inferences about the robustness of these metrics in brain tumor patients. Both LI_e_ and LI_m_ were calculated at t_s_, t_s_-, and t_s_+ to account for the threshold-dependence of LI [32,33], which can highly influence results and thus reliability. In addition, both LIs were calculated based on ROIs within the inferior frontal gyrus and the superior temporal gyrus to include Broca’s area and Wernicke’s area, respectively. Laterality was determined according to a classification system commonly used in the literature [34]: an LI value greater than 0.2 was classified as left-lateralized, less than -0.2 was classified as right-lateralized, and the remaining LI range was classified as bilateral. Reliability of language laterality was subsequently assessed by two separate methods. In method A, language laterality was deemed reliable if the same classification was produced for both test and re-test runs for LI_e_ and/or LI_m_, and across all thresholds: t_s_, t_s_-, and t_s_+. In method B, the value of individual subject LI values for the test runs was plotted against the analogous values for the re-test runs. For each functional task, the Pearson correlation coefficient r was computed to assess the consistency of the relative ordering of subject LI values within each cohort.

## Results

Based on qualitative assessment during the initial training session, all subjects complied with instructions during task performance and demonstrated competent use of the tablet. Given that some subjects performed only a subset of the behavioral tasks, a total of 12 patient datasets (6 LGG, 6 HGG) were acquired for each of the three behavioral tasks. Similarly, 12 healthy control datasets were acquired for comparison.

Subject performance scores in the test and retest runs were highly similar for both the RW task and PF task. The majority of subjects had a rhyming accuracy of >90% and generated approximately 6–13 words per 60 s task block during the PF test. HGG patients yielded some of the poorest performance scores, with rhyming accuracy falling below 80% (primarily due to slow reaction time) and only 3–4 words generated per 60 s task block of PF.

Anatomical regions of interest were shifted in one HGG patient (P02) and as a result the task ROIs were manually extended posteriorly to include the pre- and post-central gyri. The most significantly activated language areas (Table 2) in the control group maps (Fig 2) included Brodmann areas 6, 9, 22 (Wernicke’s area), 45 (Broca’s area), and 46. Group-level hand motor activity was dominated by the right precentral gyrus, however, left hemispheric activity was seen within the precentral gyrus at the single-subject level in some cases.

All but two patients (P12, P16) went on to receive surgical treatment in which fMRI data were utilized in conjunction with intraoperative brain mapping by DCS, demonstrating good concordance. The remaining patients underwent conventional surgical resection without intraoperative mapping.

### Patient vs. Controls

**Fig 3:**
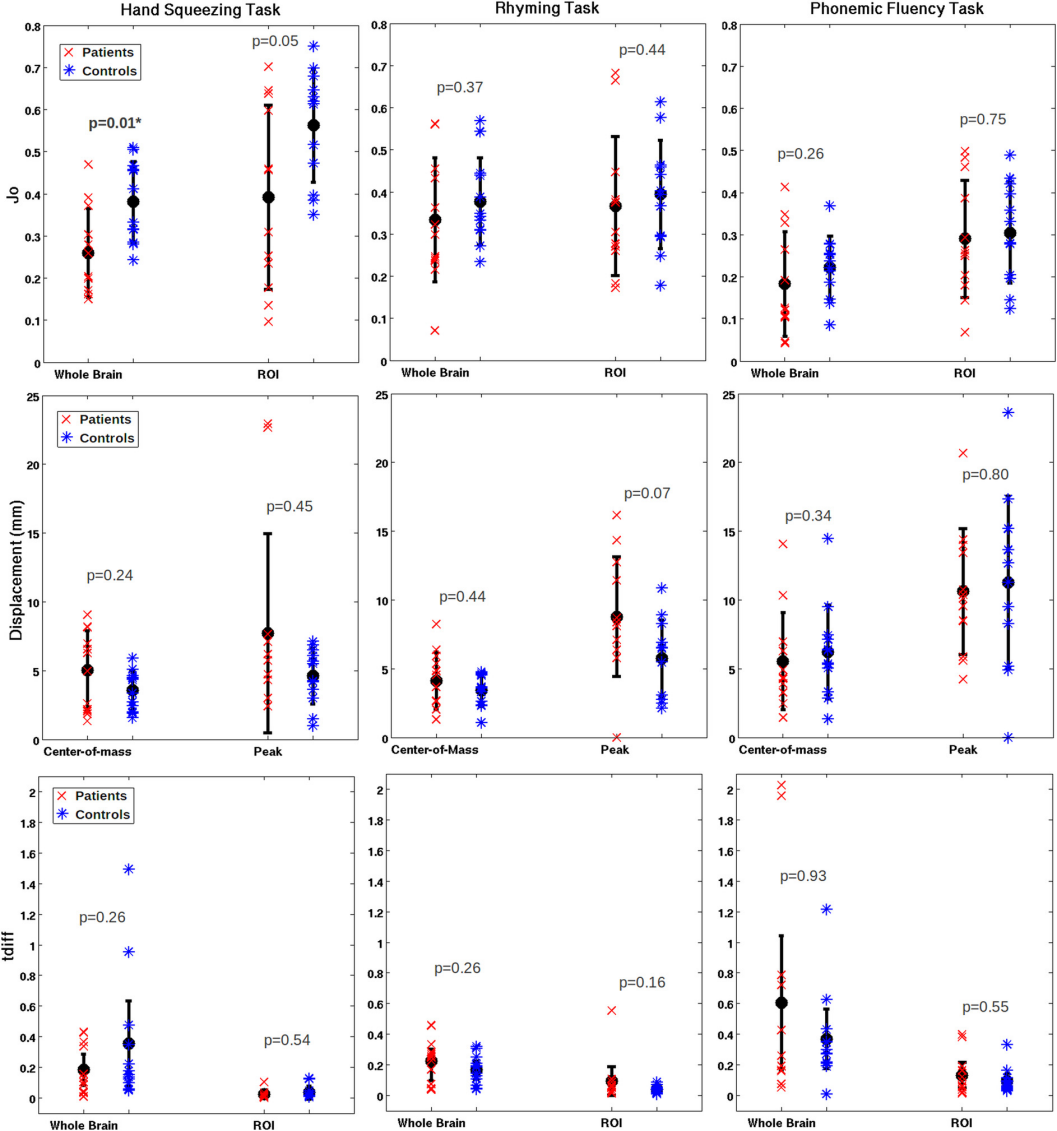
Group means (patients and controls), standard error, and single-subject values for the Jaccard index, COM/Peak displacement, and t_diff_. Significant p-values are bold and labelled with an asterisk. Note: 95% confidence intervals are plotted for t_diff_.

**Fig 4:**
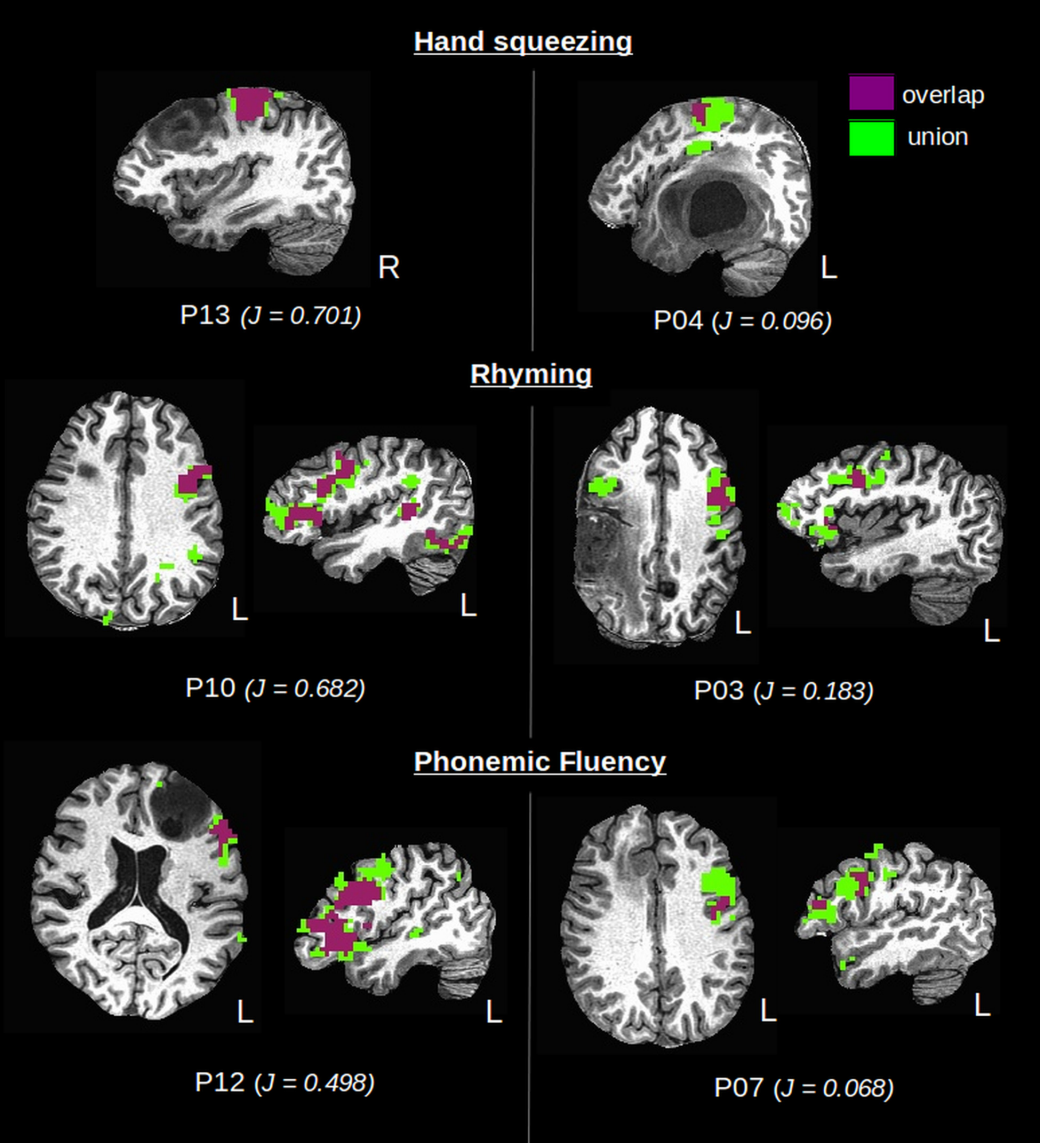
Single-subject overlap (red) and union (blue) of active voxels across test sessions. Patients who produced the highest (left column) and lowest (right column) Jaccard index (J_o_) are shown for each functional task in the left and right columns, respectively.

Group means and standard deviations for all test-retest metrics are summarized in Table 3, for patients and controls and for each fMRI task that was investigated. Fig 3 shows the metric data for individual subjects (excluding D_o,_ which produced similar trends to J_o_; and LI which is reported below). Considerable variability was observed within and between groups, and only the whole brain J_o_ (patients: 0.26±0.11, controls: 0.38±0.10) and D_o_ (patients: 0.40±0.13, controls: 0.55±0.10) showed significant group differences, specifically for the HS task. Fig 4 illustrates one aspect of the variability within the patient group, displaying overlap results for those individual patients who produced the highest and lowest Jaccard indices within each functional task.

**Table 3:** Average reliability metrics and p-values returned from the Wilcoxon rank sum test for comparisons between the patient and control groups. Significant p-values are bold and labelled with an asterisk (*).

**Hand Squeezing Task**			
*Metric*	*Patients*	*Controls*	*p-value*
Whole brain Jaccard index	0.26±0.11	0.38±0.10	0.09E-1*
ROI Jaccard index	0.39±0.22	0.56±0.13	0.05
Whole brain Dice coefficient	0.40±0.13	0.55±0.10	0.09E-1*
ROI Dice coefficient	0.53±0.23	0.71±0.12	0.05
Center-of-mass Displacement (mm)	5.81±2.85	3.57±1.43	0.24
Peak Displacement (mm)	10.60±9.60	4.60±2.04	0.45
Whole brain t_diff_	0.18±0.16	0.36±0.44	0.26
ROI t_diff_	0.02±0.03	0.04±0.04	0.54
**Phonemic Fluency Task**			
*Metric*	*Patients*	*Controls*	*p-value*
Whole brain Jaccard	0.18±0.1	0.22±0.08	0.26
ROI Jaccard	0.29±0.14	0.30±0.12	0.75
Whole brain Dice coefficient	0.29±0.17	0.36±0.10	0.26
ROI Dice coefficient	0.43±0.16	0.49±0.14	0.31
Center-of-mass Displacement (mm)	5.55±3.53	6.20±0.09	0.34
Peak Displacement (mm)	10.62±4.56	11.27±6.28	0.80
Whole brain t_diff_	0.60±0.69	0.31±0.37	0.93
ROI t_diff_	0.13±0.13	0.09±0.08	0.55
**Rhyming Task**			
*Metric*	*Patients*	*Controls*	*p-value*
Whole brain Jaccard	0.33±0.15	0.38±0.10	0.37
ROI Jaccard	0.37±0.16	0.39±0.12	0.44
Whole brain Dice coefficient	0.49±0.17	0.54±0.10	0.41
ROI Dice coefficient	0.52±0.17	0.56±0.13	0.41
Center-of-mass Displacement (mm)	4.13±2.10	3.42±1.15	0.44
Peak Displacement (mm)	8.79±4.36	5.77±2.78	0.07
Whole brain t_diff_	0.22±0.12	0.17±0.09	0.26
ROI t_diff_	0.09±0.15	0.04±0.02	0.18

Some additional trends were notable in Fig 3 for COM and peak displacement values, as well as for t_diff_ values. Apart from the PF task, where displacement values were high compared to other tasks for both patients and controls, average COM and peak displacement values for the control group were smaller (i.e. more reliable) than average measures in the patient group. The variability in COM values was smaller than for peak displacement values in both groups, and for both metrics, variability was smaller in the control group than in the patient group. Specifically, the COM displacement within the control group was well within 5 mm on average for both HS and RW tasks, with a maximum range just exceeding 5 mm. For patients, the average COM displacement was 5 mm or less across both tasks, but the maximum range was substantially larger than for controls, at approximately 8 mm. The range of observed peak displacements was particularly high in the patient group, with a maximum peak displacement value for the HS task of nearly 23 mm, and an analogous value of approximately 17 mm for the RW task. Patient and control t_diff_ values were most variable within the PF task, which showed in agreement with the RW task, that on average, t_diff_ was greater (i.e. less reliable) in patients than controls. Although the opposite trend was observed in the motor task, all tasks were consistent with respect to trends in the within- and between- group variability of t_diff_, which was smaller for ROI versus whole brain analyses.

**Fig 5:**
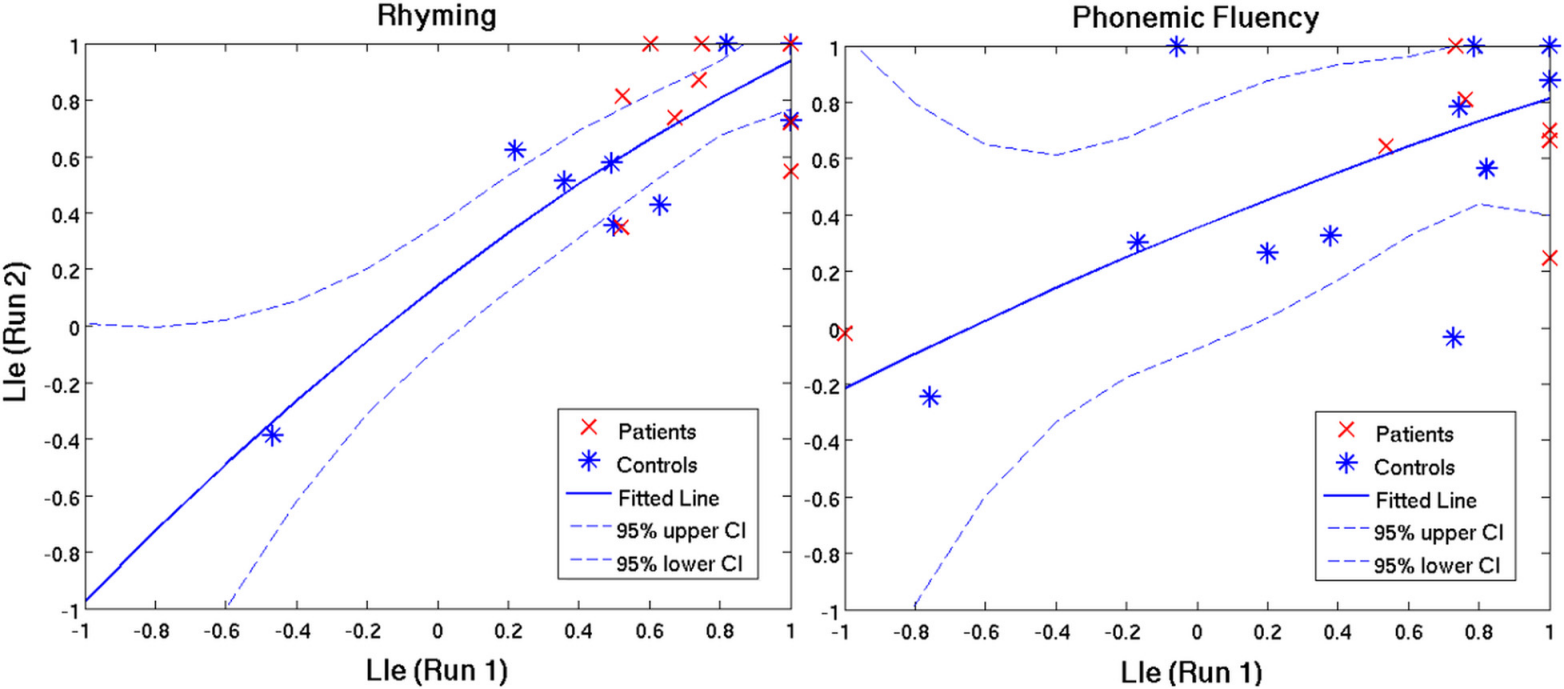
Test and re-test run correlations of LI_e_ (laterality index characterized by the extent of activated brain volume) in patients (red) and controls (blue) for the rhyming task and phonemic fluency task. The line of best fit and confidence interval (CI) pertain to the healthy control group (blue).

Based on the stringent criteria for method A (i.e. laterality consistently characterized across LI_e_, LI_m_, t_s_, t_s_-, and t_s_+), the laterality index was found to be reproducible in 92% of patients and 100% of controls for the RW task, versus a contradicting trend of 92% of patients and 67% of controls for the PF task. When LI_e_ and LI_m_ were identified as separate criteria (Table 4), percentage values increased solely for the latter metric, thus indicating better reliability when LI is measured according to brain map signal intensities. The Pearson correlation coefficients calculated in method B are tabulated in Table 4 and representative plots are illustrated in Fig 5. Similar to the findings in method A, r-values were greater (more reliable) in the controls than the patients for the RW task and *vice versa* for the PF task. However, in both the RW task and PF task results, data plotted for the individual patients fell near or within the confidence interval (CI) outlined for the control group (Fig 5), and furthermore demonstrated stronger lateralization in comparison to the controls. There was a greater amount of variability seen within the control group for the PF task versus the RW task, reflected in the larger CIs seen in Fig 5. Correlations between test and re-test run LI_e_ were minimally-to-moderately better than LI_m_ correlations, with the exception of the PF task in control subjects where the opposite effect was observed.

**Table 4:** Reliability metrics for language laterality in controls, HGG patients and LGG patients. LI_e_ and LI_m_ represent the laterality index characterized by the extent of activated brain volume, and the sum of brain map signal intensity, respectively. The Pearson correlation coefficients, r, are reported with their respective confidence interval (CI) in brackets.

**Rhyming Task**				
***Metric***	***Patients (n = 12)***	***Controls (n = 12)***	***HGG (n = 6)***	***LGG (n = 6)***
% of subjects with reproducible laterality across LI_e_, LI_m_, t_s_, t_s_-, and t_s+_	91.7% [75,100]	100%	83.3% [50,100]	100%
% of subjects with reproducible laterality across LI_e_, t_s_, t_s_-, and t_s+_	91.7% [75,100]	100%	83.3% [50,100]	100%
% of subjects with reproducible laterality across LI_m_, t_s_, t_s_-, and t_s+_	100%	100%	100%	100%
Group Pearson correlation coefficient (LI_e_ at t_s_)	r = 0.34 [-0.29,0.76]	r = 0.91 [0.72,0.98]	r = 0.11 [-0.77,0.84]	r = 0.55 [-0.47,0.94]
Group Pearson correlation coefficient (LI_m_ at t_s_)	r = -0.03 [-0.59,0.56]	r = 0.90 [0.68,0.97]	r = 0.38 [-0.62,0.91]	r = 0.11 [-0.77,0.85]
**Phonemic Fluency Task**				
***Metric***	***Patients (n = 12)***	***Controls (n = 12)***	***HGG (n = 6)***	***LGG (n = 6)***
% of subjects with reproducible laterality across LI_e_, LI_m_, t_s_, t_s_-, and t_s+_	91.7% [75,100]	66.7% [41.7,91.7]	83.3% [50,100]	100%
% of subjects with reproducible laterality across LI_e_, t_s_, t_s_-, and t_s+_	91.7% [75,100]	66.7% [41.7,91.7]	83.3% [50,100]	100%
% of subjects with reproducible laterality across LI_m_, t_s_, t_s_-, and t_s+_	91.7% [75,100]	75.0% [50,100]	83.3% [50,100]	100%
Group Pearson correlation coefficient (LI_e_ at t_s_)	r = 0.72 [0.24,0.91]	r = 0.63 [0.08,0.88]	r = 0.73 [-0.20,0.97]	r = 0.05 [-0.79,0.83]
Group Pearson correlation coefficient (LI_m_ at t_s_)	r = 0.69 [0.19,0.91]	r = 0.66 [0.14,0.90]	r = 0.72 [-0.22,0.97]	r = 0.50 [-0.52,0.93]

#### HGG vs. LGG

**Fig 6:**
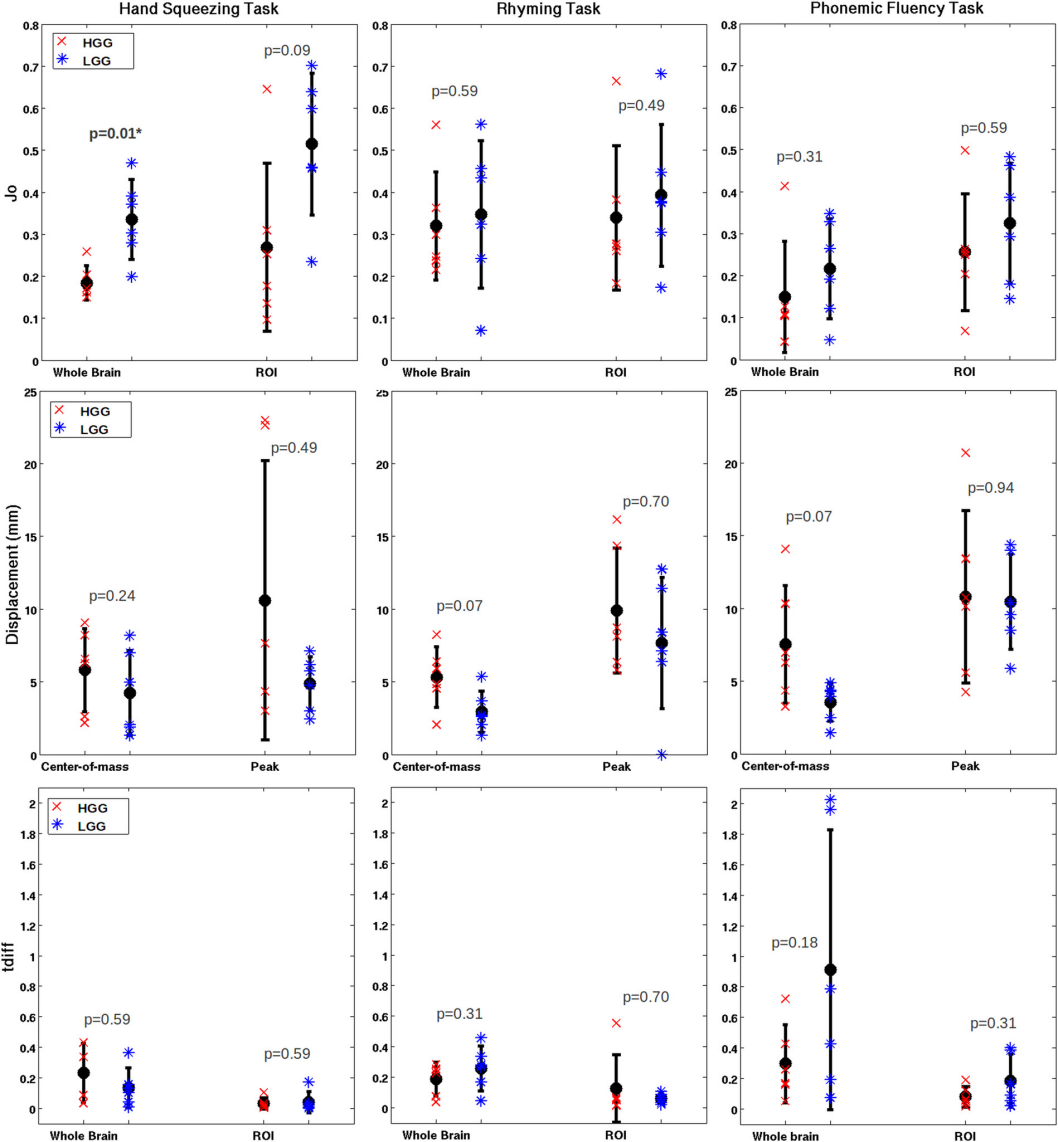
Group means (high-grade glioma = HGG and low-grade glioma = LGG), standard error, and single-subject values for the Jaccard Index, COM/Peak displacement and t_diff_. Significant p-values are bold and labelled with an asterisk. Note: 95% confidence intervals are plotted for t_diff_.

Regarding the effect of tumor grade on fMRI reliability, group means and standard deviations for all test-retest metrics are tabulated in Table 5, and plotted per subject in Fig 6 (excluding D_o_ and LI). The only statistically significant results were found for whole brain J_o_ (HGG: 0.18±0.04, LGG: 0.34±0.09) and D_o_ (HGG: 0.30±0.05, LGG: 0.49±0.09) measurements for the motor task, as similarly reported for patient-control group comparisons.

**Table 5:** Average reliability metrics and p-values returned from the Wilcoxon rank sum test for comparisons between the HGG and LGG groups. Significant p-values are bold and labelled with an asterisk (*).

**Hand Squeezing Task**			
*Metric*	*HGG*	*LGG*	*p-value*
Whole brain Jaccard index	0.18±0.04	0.34±0.09	0.09E-1*
ROI Jaccard index	0.27±0.20	0.51±0.17	0.093
Whole brain Dice coefficient	0.30±0.05	0.49±0.09	0.04E-1*
ROI Dice coefficient	0.39±0.22	0.66±0.16	0.09
Center-of-mass Displacement (mm)	5.81±2.85	4.22±2.92	0.24
Peak Displacement (mm)	10.60±9.60	4.86±1.84	0.49
Whole brain t_diff_	0.23±0.18	0.13±0.13	0.59
ROI t_diff_	0.03±0.36	0.04±0.07	0.59
**Phonemic Fluency Task**			
*Metric*	*HGG*	*LGG*	*p-value*
Whole brain Jaccard	0.15±0.13	0.22±0.12	0.31
ROI Jaccard	0.26±0.14	0.32±0.14	0.59
Whole brain Dice coefficient	0.24±0.17	0.34±0.17	0.31
ROI Dice coefficient	0.39±0.17	0.47±0.16	0.59
Center-of-mass Displacement (mm)	7.54±4.02	3.56±1.32	0.65
Peak Displacement (mm)	10.79±5.92	10.44±3.27	0.94
Whole brain t_diff_	0.29±0.24	0.91±0.87	0.18
ROI t_diff_	0.08±0.06	0.19±0.17	0.31
**Rhyming Task**			
*Metric*	*HGG*	*LGG*	*p-value*
Whole brain Jaccard	0.32±0.13	0.35±0.18	0.59
ROI Jaccard	0.34±0.17	0.39±0.17	0.49
Whole brain Dice coefficient	0.48±0.14	0.50±0.21	0.59
ROI Dice coefficient	0.49±0.17	0.55±0.17	0.49
Center-of-mass Displacement (mm)	5.32±2.07	2.94±1.42	0.65
Peak Displacement (mm)	9.91±4.31	7.67±4.49	0.70
Whole brain t_diff_	0.19±0.10	0.26±0.14	0.31
ROI t_diff_	0.13±0.21	0.06±0.03	0.70

There were consistent trends for mean J_o_ and D_o_ values across all tasks, showing that mean overlap for HGG patients is reduced slightly compared to LGG patients. The COM and peak displacement also had consistent trends across tasks, showing that HGG patients had slightly poorer reliability based on these metrics. However, the t_diff_ metric did not show consistent trends across tasks. For example, in Fig 6 the rhyming task demonstrated a reduced t_diff_ (poorer reliability) for HGG patients compared to LGG patients when analyzed across the whole brain, yet an increased t_diff_ (better reliability) for HGG patients when analyzed within the ROI.

The laterality index was reproducible in 100% of LGG patients versus 83.3% of HGG patients (Table 4) for both the RW task and PF task, at the most stringent reliability criteria. When LI_m_ was considered separately from LI_e_, the classification of laterality was improved in one HGG patient for the RW task. Trends in the Pearson correlation coefficients, however, were inconsistent and contradictory to trends identified through method A.

### Motor vs. Language

Although the effects across patients, controls, and tumor grade were primarily identified through trends in the data, significant effects were observed across tasks, though fewer in the patient group (Table 6). Spatial overlap measures were on average comparable for the HS and RW task, whereas results for the PF task were significantly lower. For example, in the control group, a Jaccard index (whole brain) of 0.38±0.10 was reported for both the HS and RW task, versus a Jaccard index of 0.22±0.08 for the PF task. COM and peak displacement values were also comparable for the HS task and RW task, whereas the values were doubled or nearly doubled for PF, associated with a much poorer reliability outcome. Statistically significant differences in t_diff_ were identified in both comparisons (i.e. HS vs. RW, HS vs. PF), solely for ROI measurements.

**Table 6:** Group means, standard deviations and p-values returned from the Wilcoxon rank sum test for comparisons between the RW, PF, and HS tasks in both patients and controls. Significant p-values are bold and labelled with an asterisk (*).

**Hand Squeezing Task vs. Rhyming Task**						
	***Patients***			***Controls***		
***Metric***	***HS***	***RW***	***p-value***	***HS***	***RW***	***p-value***
Whole brain Jaccard	0.26±0.11	0.33±0.15	0.18	0.38±0.10	0.38±0.10	0.84
ROI Jaccard	0.39±0.22	0.37±0.16	0.99	0.56±0.13	0.39±0.12	0.09E-1*
Whole brain Dice coefficient	0.40±0.13	0.49±0.17	0.13	0.55±0.10	0.54±0.10	0.93
ROI Dice coefficient	0.53±0.23	0.52±0.17	1.00	0.71±0.12	0.56±0.13	0.01*
Center-of-mass Displacement (mm)	5.81±2.85	4.13±2.10	0.54	3.57±1.43	3.42±1.15	0.98
Peak Displacement (mm)	10.60±9.60	8.79±4.36	0.10	4.60±2.04	5.77±2.78	0.40
Whole brain t_diff_	0.18±0.16	0.22±0.12	0.51	0.36±0.44	0.17±0.09	0.47
ROI t_diff_	0.02±0.03	0.09±0.15	0.09E-1*	0.04±0.04	0.04±0.02	0.16
**Hand Squeezing Task vs. Phonemic Fluency Task**						
***Metric***	***HS***	***PF***	***p-value***	***HS***	***PF***	***p-value***
Whole brain Jaccard	0.26±0.11	0.18±0.1	0.07	0.38±0.10	0.22±0.08	3.84E-4*
ROI Jaccard	0.39±0.22	0.29±0.14	0.40	0.56±0.13	0.30±0.12	9.01E-4*
Whole brain Dice coefficient	0.40±0.13	0.29±0.17	0.08	0.55±0.10	0.36±0.10	2.46E-4*
ROI Dice coefficient	0.53±0.23	0.43±0.16	0.34	0.71±0.12	0.49±0.14	0.02E-1*
Center-of-mass Displacement	5.81±2.85	5.55±3.53	0.98	3.57±1.43	6.20±0.09	0.17E-1*
Peak Displacement	10.60±9.60	10.62±4.56	0.46E-1*	4.60±2.04	11.27±6.28	0.06E-1*
Whole brain t_diff_	0.18±0.16	0.60±0.69	0.05	0.36±0.44	0.31±0.37	0.26
ROI t_diff_	0.02±0.03	0.13±0.13	0.02E-1*	0.04±0.04	0.09±0.08	0.06E-1*
**Rhyming Task vs. Phonemic Fluency Task**						
***Metric***	***RW***	***PF***	***p-value***	***RW***	***PF***	***p-value***
Whole brain Jaccard	0.33±0.15	0.18±0.1	0.26E-1*	0.38±0.10	0.22±0.08	9.01E-4*
ROI Jaccard	0.37±0.16	0.29±0.14	0.31	0.39±0.12	0.30±0.12	0.11
Whole brain Dice coefficient	0.49±0.17	0.29±0.17	0.23E-1*	0.54±0.10	0.36±0.10	5.92E-4*
ROI Dice coefficient	0.52±0.17	0.43±0.16	0.29	0.56±0.13	0.49±0.14	0.31
Center-of-mass Displacement (mm)	4.13±2.10	5.55±3.53	0.44	3.42±1.15	6.20±0.09	0.09E-1*
Peak Displacement (mm)	8.79±4.36	10.62±4.56	0.44	5.77±2.78	11.27±6.28	0.19E-1*
Whole brain t_diff_	0.22±0.12	0.60±0.69	0.26	0.17±0.09	0.31±0.37	0.17E-1*
ROI t_diff_	0.09±0.15	0.13±0.13	0.34	0.04±0.02	0.09±0.08	0.14E-1*

### Rhyming vs. Phonemic Fluency

Spatial overlap, displacement, and t_diff_ metrics consistently demonstrated better reliability outcomes for the RW task than for the PF task (Table 6). Statistically significant differences between tasks for J_o_ and D_o_ were restricted to whole brain measures in both patients and controls, whereas significant results were limited to the control group for the displacement and t_diff_ metrics. The classification of language laterality (i.e. left, right, or bi-lateral) was equally reproducible in patients across tasks, but in the control group, language laterality was reproducible in a greater number of subjects for the RW task. Additionally, LI values for test and re-test runs were better correlated in the RW task for controls (Fig 5 and Table 4); however, this was contradicted in the patient group.

## Discussion & Conclusions

Functional MRI is gaining popularity in clinical applications such as preoperative planning for brain tumor surgery, but it is very important that the brain activity maps derived from the fMRI BOLD signal are of sufficient quality to support their intended use. The main method for assessing fMRI quality is to perform test-retest studies and analyze the resulting data in terms of various reliability metrics that quantify the constancy of the measured signals. Because the fMRI test-retest literature focuses predominantly on healthy controls, the present study was undertaken to fill a gap in existing knowledge about clinically-relevant fMRI data quality by assessing multiple reliability metrics in a cohort of brain tumor patients. This included the use of single-subject optimization of the fMRI preprocessing pipeline to generate robust results, division of the patient group to study the influence of tumor grade on fMRI reliability metrics, and comparison with patient-matched healthy controls under identical behavioral testing conditions across three different tasks for mapping motor and language regions.

### Patients & Controls

The main finding from this study is that for the most part, fMRI reliability metrics for the sample population of brain tumor patients are very comparable to those for healthy controls. It was demonstrated that reliability metric outcomes for both patients and controls are highly variable and dependent on the tumor grade in patients, choice of behavioral task, reliability metric, and the level of analysis (whole brain versus ROI). Each of these factors is discussed subsequently; however, the effect of tumor grade is given precedence due to the novelty of the finding. Specifically, fMRI reliability metrics were slightly worse on average for HGG patients when compared to those for LGG patients. In addition, the reliability metrics for LGG patients and controls were very similar. The broad implication is thus that the characteristics of healthy control fMRI test-retest data are very likely applicable to LGG patients, and that previous findings from the literature can be used to make inferences about reliability for preoperative planning in patients with low-grade tumors. This is important, given that the growth industry for preoperative fMRI is being driven in part by an evolving clinical predisposition toward early intervention for LGG.

The differences in fMRI reliability metrics observed between HGG and LGG patients are consistent with two factors influencing fMRI signals that have been previously described in the literature. It has been reported that tumor-induced neurovascular uncoupling (ti-NVU) of the BOLD signal is more pronounced in HGG than in LGG, due to increased hyperperfusion of the vasculature [35]. The ti-NVU effect is anticipated to have most impact as the LAD approaches zero in high-grade tumors, where large volume effects are often exhibited. The second important factor involves the influence of substantial preoperative neurological deficit in patients with high-grade tumors (Table 1), resulting in poorer execution of fMRI tasks or fatigue on test-retest experiments. In particular, clinical data show significant correlations between HGG and low Karnofsky Performance Scores [36].

Although both of these factors are likely to have influenced the results of this study to some extent, their importance and relative contributions are difficult to ascertain in practice because of the experimental design and patient variability. Interestingly, no obvious relationship was detected between fMRI reliability metrics and LAD, or between reliability metrics and fMRI performance scores. Fig 4 clearly demonstrates examples where the most reliable activation maps (as measured by overlap of activation clusters) were obtained immediately adjacent to LGG tumors (P12, P13), but also examples where overlap was the worst for activations in the hemisphere contralateral to the LGG tumor site (P03). Regarding fMRI performance scores, HGG patients did produce some of the lowest performances, but they were highly patient-dependant and highly uncorrelated with reliability outcomes. For example, one patient (P06) who presented with a grade III anaplastic astrocytoma, performed at a high level on all fMRI tasks, producing up to 13 words per minute in the PF task. Yet, reliability metric outcomes remained poor for this subject and were significantly lower than average results for LGG and controls. Tumor stage was found to be the only strong predictor of fMRI reliability metrics: the HGG patient group consistently yielded slightly worse metrics on average in comparison to the LGG group, regardless of between-patient variations in LAD or performance scores.

Putting the above discussion in context, it is also important to emphasize that the sample size of the present data poses a limitation to understanding more fully the relationship between fMRI reliability and tumor grade. Further investigation of this relationship in a larger cohort of patients is left to future work. Nonetheless, the present results provide strong enough evidence to suggest that clinicians should use some additional level of caution when interpreting activation maps in patients with suspected high-grade tumors. Preoperative evaluation of these patients is crucial such that the neuroimaging technician is aware of any language or motor deficits, and can improve reliability of the data by repeating fMRI paradigms that engage the compromised brain networks.

### Behavioral Tasks

Differences in fMRI reliability metrics between patients and controls were evaluated for three different behavioral tasks: a hand squeezing task, a forced-choice rhyming task, and a written phonemic fluency task. Subject performance scores agree well with previous findings in the literature. For example, Golestanirad *et al*. (2015) reported a similar finding for the written PF task using the tablet system, where subjects generated an average of 12.1 ± 2.7 words per minute across all letters (F, A, S, D, or C) [21]. Zec *et al*. (1999) also found similar results with overt speech, reporting an average of 12.2±4.8, 11.0±5.0, and 13.4±4.6 words respectively for letters F, A, and S [37]. Group maps generated for each task were also consistent with previous literature, based on regions of peak brain activity (Table 2 and Fig 2) [20,21]. A statistically significant difference in group means, driven primarily by the HGG patients, was found solely for the hand squeezing task. This task yielded the most reliable activation maps, in agreement with previous findings in the literature suggesting better reliability for motor versus language tasks [11,38]. Language task differences in fMRI reliability metrics between patients and controls lacked statistical significance, consistent with the high inter-subject variability previously observed for tasks that have higher cognitive components than simple motor components [39]. This effect is further supported by the observation that fMRI reliability metrics for the HS and RW tasks were much less different than those for hand squeezing and the PF task. The cognitive demands including visual scanning, sustained attention, constrained response selection, and language processing were relatively modest for the RW task. In contrast, the written PF task is a measure of executive function, with higher cognitive demands including mental flexibility, unconstrained search for mental lexicon, and written coordination [20]. The unconstrained nature of the task means that performance depends on multiple factors including the size of the working lexicon and the ability to swap between clusters of similar word types once a cluster is depleted. Furthermore, the task becomes more difficult over time as fluency decreases, with consequent changes in brain activity that can reduce fMRI signals and lead to poorer reliability [21]. There is evidence in the literature of better reliability for the RW task versus the PF task [20], including: easier quantification of behavioral performance, more focal activation patterns, and stronger hemispheric lateralization for determining language laterality. Nonetheless, apart from their differences, similar peri-Sylvian activation patterns have been reported for the RW and PF tasks (Fig 2) [20]. This is valuable information for researchers and clinicians conducting fMRI studies in patients with brain tumors, for example, who may have neurological deficits that affect their performance capabilities. A task of lower cognitive difficulty (i.e. RW) likely can be used to produce a desired activation pattern in a more robust and reliable manner than a task that is more cognitively challenging (i.e. PF) and thus prone to poorer performance scores and noisier brain activation signals. In any event, multiple tasks with overlapping activation patterns should be applied during preoperative fMRI to avoid misidentification of eloquent regions. However, it could be that the PF task solely provides confirmatory evidence.

### Reliability Metrics & Level of Analysis

To quantify fMRI test-retest reliability in a comprehensive manner, the spatial overlap (via Jaccard index and Dice coefficient) of active clusters, the COM and peak displacement of active clusters, the stability of the BOLD signal across test sessions (via t_diff_) and the laterality index were investigated in the present study. The spatial overlap results agreed well with previous literature. For example, an average Dice coefficient of 45% and a Jaccard index of 33% has been reported in healthy subjects [11], and a range from 23–100% in patients with low-grade neoplasms. COM and peak displacement measures also fell within the range of what has been previously reported for healthy subjects [40,41] and patients [28,42], and the between session t-value variance, t_diff_, followed trends reported by Gorgolewski et al. (2013) [30].

Results were highly variable depending on the choice of metric as well as the level of analysis (i.e. whole brain vs. ROI). Spatial overlap measures showed the most stable trends in the data. The Dice coefficient and Jaccard index were highly correlated, differing primarily in magnitude. Although the Dice coefficient has been reported more extensively in the literature, it has been argued that the Jaccard index is a more suitable metric providing a more natural quantification of overlap [29,43].

Previous studies have reported that ROI analyses yield higher reproducibility metrics than whole brain analyses [14,30,44], similar to the results reported here for spatial overlap and t_diff_ measures. This is likely due to the removal of unstable and/or false positive activations when an ROI is constructed, however reliability remains highly dependent on appropriate ROI selection [31]. In some cases, however, the use of ROI analysis may have a marginal influence on the outcome of one reliability metric in comparison to another. This effect is seen to an extent with the J_o_ and t_diff_ metrics, where the ROI analysis had a greater influence on the former (Figs 3 and 6).

The displacement of COM coordinates was found to be more reproducible than peak coordinates of activity; previous studies reported similar findings noting the high variability in peak coordinates [42]. In fact, the peak coordinates of clusters varied as high at 23 mm in the present patient group, and Wurnig *et al*. (2013) reported measures as high as 45 mm. This poses a problem for clinicians who are often naturally inclined to look for regions of peak activity in a color-coded fMRI activation map, absent of any COM cursor data. Although the COM of a given cluster may be more reliable, it does not necessarily mean that it is representative of the true spatial coordinates of neural activity. Thus, an investigation of fMRI concordance with intraoperative mapping data, on the basis of peak versus COM coordinates, would be a valuable contribution to this field.

Ruff *et al*. (2008) evaluated the reproducibility of the LI across three different language tasks and a range of p-values, concluding that language laterality by fMRI is threshold- and task-dependent [32]. The latter was similarly shown by Nadkarni *et al*. (2015) who explored laterality within expressive versus receptive language tasks [45]. The present study found that fMRI reliability has a task-dependence. Better correlations between individual test and re-test LIs were found for the RW task in comparison to the PF task, with the former used much more frequently to determine lateralization by fMRI [46,47]. Nonetheless, for both the RW task and PF task, laterality was classified as left, right, or bi-lateral, consistently across thresholds in a high number of patients (11/12 or 92%). This supports previous data which have shown high concordance between fMRI language lateralization and intraoperative mapping data [48]. Together these data suggest that fMRI is suitable for determining language laterality in brain tumor patients, provided that the methods for calculating LI are standardized. Although no formal methods have been outlined, some have proposed the use of functionally driven ROIs; as well as multiple cognitive tasks, thresholds, and definitions pertinent to LI (e.g. LI_e_, LI_m_) [31].

Although not explicitly evaluated here, the choice of statistical threshold used to map brain activity is an important factor that influences fMRI reliability. For example, McKinsey *et al*. (2010) demonstrated in a group of 24 brain tumor patients that deviations of ±20% from a standard fMRI threshold (t ranging from 2.8 to 26.4 across subjects) had no significant effect on COM, number of activated voxels, or the reproducibility of the location of activated voxels [28]. More recently, Stevens *et al*. (2013) conducted data-driven analyses in 8 patients, reporting a decrease in spatial overlap of approximately 10% when the threshold was varied from t = 2.6 to t = 6.6 [49]. It is worth noting however, that the rate of decrease slowed with increasingly higher fMRI thresholds, and individual results were highly variable. Irrespective of the slightly different findings in the literature, the potential volatility of fMRI thresholds is widely appreciated as a concern. The present study has addressed the issue indirectly through use of individually-optimized preprocessing pipelines, and evaluation of reliability metrics s across t_s_, t_s_-, and t_s_+, as reasonable attempts towards minimizing within-subject effects and ensuring between-subject variability has more influence on reported reliability measures.

### Data Preprocessing Strategies

Preprocessing strategies were implemented into the analysis to mitigate subject-specific artifacts, including physiological noise and head motion that is likely more pronounced in a patient population. Churchill *et al*. (2013) demonstrated using the NPAIRS framework that single-subject pipeline choices can significantly affect data outcomes, and that the use of individually-optimized pipelines over standard pipelines improves fMRI reliability [15,16]. Thus, their algorithm was applied to single-subject data, thereby controlling for the above mentioned artifacts while allowing other confounding factors (e.g. brain tumor) to be addressed. However, this is not the only reliable method that has been reported in the literature. In addition to the NPAIRS framework, others have used empirical receiver operating characteristic (ROC) analyses to optimize single-subject pipelines [50,51]. Recently, Stevens *et al*. (2015) reported a significant increase in fMRI reliability for brain tumor patients using an ROC-based novel approach to select optimal pipeline choices [38]. Average reliability measures in their patient group increased from 0.58±0.03 to 0.65±0.02 through the use of optimized preprocessing pipelines, thereby narrowing the gap in reliability outcomes between the patients and a cohort of healthy controls (0.72±0.02).

As such, there are significant benefits to adopting one of such methods when dealing with clinical populations where inter-subject variability is high. However, it is also worth noting that the NPAIRS optimal preprocessing pipelines selected for patients and controls were carefully scrutinized and found to have no clear and consistent differences. This suggests that the variability attributable to fMRI signal artifact is comparable in both groups and that pipeline optimization is of general benefit, rather than a benefit solely in patient populations.

### Clinical Relevance & Suitability

Quantitative measures of reliability are primarily used to validate fMRI for preoperative planning, however to properly assess its validity, a discussion of the clinical relevance is required. One very important question to consider when interpreting the results is where fMRI reliability is most important to the surgeon. In the majority of test-retest studies, reliability is measured across the whole brain and functionally driven ROIs, while in clinical practice the surgeon is only concerned with the regions immediately adjacent to the tumor that are exposed in the craniotomy window. Thus, it is the reliability of those activations adjacent to or nearby the tumor that are of clinical relevance. In Fig 4, examples were provided in LGG patients where activations adjacent to the tumor demonstrated good reliability results via the Jaccard index and Dice coefficient. Although reliability might be good in some patients, the variability across subjects remains high and there is a need for practical thresholds to help determine the acceptable measure of reliability in a real clinical situation.

Functional MRI maps may be a valuable adjunct to determine the craniotomy extent, direct targets for DCS, and perform subcortical mapping. In a worst-case scenario, the consequences of reliability may be that an fMRI finding causes the surgeon to expose more of the brain than necessary, or that the surgeon expends a few minutes of additional surgical time to perform subcortical mapping. Here, fMRI serves as a guide for planning; DCS remains the gold standard used to direct the surgical process in real-time [52–56].

In addition to characterizing fMRI reliability, it is also very important to carefully evaluate how well fMRI and DCS results agree. Overall, the neurosurgical opinion was that fMRI and DCS were in good agreement for each of the patients studied here, and that fMRI made valuable contributions to surgical decisions as a consequence. A more detailed and quantitative analysis of these sentiments is beyond the scope of the present work. Similar to fMRI, DCS remains an imperfect technique and therefore there is a broad need to understand and quantify the error introduced by various sources of technical and biological variability that may influence concordance between DCS and fMRI results. Our laboratory intends to report on these issues in the very near future.

Results from this study suggest that preoperative fMRI is a suitable clinical tool for patients diagnosed with LGG, whereas reliability decreases somewhat for patients with HGG. Further study of fMRI reliability in HGG patients will be useful. The choice of behavioral task, reproducibility metric, and level of analysis all influence fMRI reliability and whether differences can be observed between patients and healthy controls. Although the demands of fMRI reliability may be lessened when the clinical consequences of false information in fMRI maps are less severe to the patient, quantitative thresholds for reliability in LGG and HGG patients are of value and obtainable. Toward this goal, the test-retest data presented here must be augmented by additional patient recruitment, and further validated by intraoperative brain mapping data. This is the subject of on-going work in our laboratory, in an attempt to establish a flexible threshold for reliability that will ease the use of fMRI in practice, enabling it to be applied confidently in the clinical decision-making process.

## Abbreviations

BOLD: blood oxygenation level-dependent
CI: confidence interval
COM: center of mass
DCS: direct cortical stimulation
FOV: field of view
HGG: high grade glioma
HS: hand squeezing
LAD: lesion-to-activation distance
LGG: low grade glioma
LI: laterality index
PF: phonemic fluency
RW: rhyming words
TE: echo time
TR: repetition time
ti-NVU: tumor-induced neurovascular uncoupling
WHO: world health organization

## References

[1] Sunaert S. Presurgical planning for tumor resectioning. J Magn Reson Imaging. 2006;23: 887–905. 10.1002/jmri.20582 16649210

[2] Detre J a. fMRI: applications in epilepsy. Epilepsia. 2004;45 Suppl 4: 26–31. 10.1111/j.0013-9580.2004.04006.x 15281954

[3] Detre JA. Clinical applicability of functional MRI. J Magn Reson Imaging. 2006;23: 808–815. 10.1002/jmri.20585 16649200

[4] Wilkinson ID, Romanowski CAJ, Jellinek DA, Morris J, Griffiths PD. Motor functional MRI for pre-operative and intraoperative neurosurgical guidance. Br J Radiol. 2003;76: 98–103. 10.1259/bjr/66817309 12642277

[5] Janecek JK, Swanson SJ, Sabsevitz DS, Hammeke TA, Raghavan M, E. Rozman M, et al Language lateralization by fMRI and Wada testing in 229 patients with epilepsy: Rates and predictors of discordance. Epilepsia. 2013;54: 314–322. 10.1111/epi.12068 23294162 PMC3649863

[6] Rutten GJM, Ramsey NF, Van Rijen PC, Noordmans HJ, Van Veelen CWM. Development of a functional magnetic resonance imaging protocol for intraoperative localization of critical temporoparietal language areas. Ann Neurol. 2002;51: 350–360. 10.1002/ana.10117 11891830

[7] Petrella JR, Shah LM, Harris KM, Friedman AH, George TM, Sampson JH, et al Preoperative functional MR imaging localization of language and motor areas: effect on therapeutic decision making in patients with potentially resectable brain tumors. Radiology. 2006;240: 793–802. 10.1148/radiol.2403051153 16857981

[8] Tieleman A, Deblaere K, Van Roost D, Van Damme O, Achten E. Preoperative fMRI in tumour surgery. Eur Radiol. 2009;19: 2523–2534. 10.1007/s00330-009-1429-z 19430795

[9] Bennett CM, Miller MB. fMRI reliability: influences of task and experimental design. Cogn Affect Behav Neurosci. 2013;13: 690–702. 10.3758/s13415-013-0195-1 23934630

[10] Seto E, Sela G, McIlroy WE, Black SE, Staines WR, Bronskill MJ, et al Quantifying head motion associated with motor tasks used in fMRI. Neuroimage. 2001;14: 284–97. 10.1006/nimg.2001.0829 11467903

[11] Bennett CM, Miller MB. How reliable are the results from functional magnetic resonance imaging? Ann N Y Acad Sci. 2010;1191: 133–155. 10.1111/j.1749-6632.2010.05446.x 20392279

[12] Raemaekers M, Du Plessis S, Ramsey NF, Weusten JMH, Vink M. Test-retest variability underlying fMRI measurements. Neuroimage. Elsevier Inc.; 2012;60: 717–727. 10.1016/j.neuroimage.2011.11.061 22155027

[13] Harrington GS, Buonocore MH, Tomaszewski Farias S. Intrasubject reproducibility of functional MR imaging activation in language tasks. Am J Neuroradiol. 2006;27: 938–944. 27/4/938 [pii]. 16611797 PMC8133984

[14] Caceres A, Hall DL, Zelaya FO, Williams SCR, Mehta MA. Measuring fMRI reliability with the intra-class correlation coefficient. Neuroimage. Elsevier Inc.; 2009;45: 758–768. 10.1016/j.neuroimage.2008.12.035 19166942

[15] Churchill NW, Oder A, Abdi H, Tam F, Lee W, Thomas C, et al Optimizing preprocessing and analysis pipelines for single-subject fMRI. I. Standard temporal motion and physiological noise correction methods. Hum Brain Mapp. 2012;33: 609–627. 10.1002/hbm.21238 21455942 PMC4898950

[16] Strother SC, Anderson J, Hansen LK, Kjems U, Kustra R, Sidtis J, et al The quantitative evaluation of functional neuroimaging experiments: the NPAIRS data analysis framework. Neuroimage. 2002;15: 747–771. 10.1006/nimg.2001.1034 11906218

[17] Tam F, Churchill NW, Strother SC, Graham SJ. A new tablet for writing and drawing during functional MRI. Hum Brain Mapp. 2012;33: 1750–1751. 10.1002/hbm.21375 PMC6870006 20336688

[18] Morrison MA, Tam F, Garavaglia MM, Golestanirad L, Hare GMT, Cusimano MD, et al A novel tablet computer platform for advanced language mapping during awake craniotomy procedures. J Neurosurg. 2015; 1–7. 10.3171/2015.4.JNS15312 26473779

[19] Glover GH, Law CS. Spiral-in/out BOLD fMRI for increased SNR and reduced susceptibility artifacts. Magn Reson Med. 2001;46: 515–522. 10.1002/mrm.1222 11550244

[20] Lurito JT, Kareken DA, Lowe MJ, Chen SHA, Mathews VP. Comparison of rhyming and word generation with FMRI. Hum Brain Mapp. 2000;10: 99–106. 10.1002/1097-0193(200007)10:3<99::AID-HBM10>3.0.CO;2-Q 10912589 PMC6872040

[21] Golestanirad L, Das S, Schweizer TA, Graham SJ. A preliminary fMRI study of a novel self-paced written fluency task: observation of left-hemispheric activation, and increased frontal activation in late vs. early task phases. Front Hum Neurosci. 2015;9: 113 10.3389/fnhum.2015.00113 25805984 PMC4354285

[22] Lezak M, Howieson D, Bigler ED, Daniel T. Neuropsychological Assessment. Oxford University Press; 2004.

[23] Cox RW. AFNI: Software for Analysis and Visualization of Functional Magnetic Resonance Neuroimages. Comput Biomed Res. 1996;29: 162–173. 10.1006/cbmr.1996.0014 8812068

[24] Campbell KL, Grigg O, Saverino C, Churchill N, Grady CL. Age differences in the intrinsic functional connectivity of default network subsystems. Front Aging Neurosci. 2013;5: 1–12. 24294203 10.3389/fnagi.2013.00073 PMC3827623

[25] Carbonell F, Bellec P, Shmuel A. Global and system-specific resting-state fMRI fluctuations are uncorrelated: principal component analysis reveals anti-correlated networks. Brain Connect. 2011;1: 496–510. 10.1089/brain.2011.0065 22444074 PMC3604782

[26] Churchill NW, Strother SC. PHYCAA+: An optimized, adaptive procedure for measuring and controlling physiological noise in BOLD fMRI. Neuroimage. Elsevier Inc.; 2013;82: 306–325. 10.1016/j.neuroimage.2013.05.102 23727534

[27] Genovese CR, Roeder K, Wasserman L. False discovery control with p -value weighting. Biometrika. 2006;93: 509–524.

[28] Mckinsey RD, Mortitz CH, Meyerand ME, Tome WA. Assessment of Multiple Task Activation and Reproducibility in Patients with Benign and Low- grade Neoplasms. Technol Cancer Res Treat. 2015;9: 319–326. 10.1177/153303461000900402 PMC2906819 20626198

[29] Maitra R. A re-defined and generalized percent-overlap-of-activation measure for studies of fMRI reproducibility and its use in identifying outlier activation maps. Neuroimage. Elsevier Inc.; 2010;50: 124–135. 10.1016/j.neuroimage.2009.11.070 19963068

[30] Gorgolewski KJ, Storkey AJ, Bastin ME, Whittle I, Pernet C. Single subject fMRI test-retest reliability metrics and confounding factors. Neuroimage. Elsevier Inc.; 2013;69: 231–243. 10.1016/j.neuroimage.2012.10.085 23153967

[31] Jansen A, Menke R, Sommer J, F??rster AF, Bruchmann S, Hempleman J, et al The assessment of hemispheric lateralization in functional MRI-Robustness and reproducibility. Neuroimage. 2006;33: 204–217. 10.1016/j.neuroimage.2006.06.019 16904913

[32] Ruff IM, Petrovich Brennan NM, Peck KK, Hou BL, Tabar V, Brennan CW, et al Assessment of the language laterality index in patients with brain tumor using functional MR imaging: Effects of thresholding, task selection, and prior surgery. Am J Neuroradiol. 2008;29: 528–535. 10.3174/ajnr.A0841 18184849 PMC8118874

[33] Rutten GJM, Ramsey NF, van Rijen PC, van Veelen CWM. Reproducibility of fMRI-determined language lateralization in individual subjects. Brain Lang. 2002;80: 421–437. 10.1006/brln.2001.2600 11896650

[34] Seghier ML. Laterality index in functional MRI: methodological issues. Magn Reson Imaging. 2008;26: 594–601. 10.1016/j.mri.2007.10.010 18158224 PMC2726301

[35] Hou BL, Bradbury M, Peck KK, Petrovich NM, Gutin PH, Holodny AI. Effect of brain tumor neovasculature defined by rCBV on BOLD fMRI activation volume in the primary motor cortex. Neuroimage. 2006;32: 489–497. 10.1016/j.neuroimage.2006.04.188 16806983

[36] Action A. Gliomas: New insight for the healthcare professionals Scholarly Editions; 2013.

[37] Zec RF, Landreth ES, Fritz S, Grames E, Hasara A, Fraizer W, et al A comparison of phonemic, semantic, and alternating word fluency in Parkinson’s disease. Arch Clin Neuropsychol. 1999;14: 255–264. 10.1016/S0887-6177(98)00008-0 14590594

[38] Stevens MTR, Clarke DB, Stroink G, Beyea SD, D’Arcy RC. Improving fMRI reliability in presurgical mapping for brain tumours. J Neurol Neurosurg Psychiatry. 2015; 1–8. 25814491 10.1136/jnnp-2015-310307

[39] Seghier ML, Lazeyras F, Pegna AJ, Annoni JM, Zimine I, Mayer E, et al Variability of fMRI activation during a phonological and semantic language task in healthy subjects. Hum Brain Mapp. 2004;23: 140–155. 10.1002/hbm.20053 15449358 PMC6871802

[40] Stringer EA, Chen LM, Friedman RM, Gatenby C, Gore JC. Differentiation of somatosensory cortices by high-resolution fMRI at 7T. Neuroimage. Elsevier Inc.; 2011;54: 1012–1020. 10.1016/j.neuroimage.2010.09.058 20887793 PMC4270280

[41] Fesl G, Braun B, Rau S, Wiesmann M, Ruge M, Bruhns P, et al Is the center of mass (COM) a reliable parameter for the localization of brain function in fMRI? Eur Radiol. 2008;18: 1031–1037. 10.1007/s00330-008-0850-z 18228024

[42] Wurnig MC, Rath J, Klinger N, Höllinger I, Geissler A, Fischmeister FP, et al Variability of clinical functional MR imaging results: a multicenter study. Radiology. 2013;268: 521–31. 10.1148/radiol.13121357 23525207

[43] Ruddell SJS, Twiss SD, Pomeroy PP. Measuring opportunity for sociality: quantifying social stability in a colonially breeding phocid. Anim Behav. 2007;74: 1357–1368. 10.1016/j.anbehav.2007.01.024

[44] Brandt DJ, Sommer J, Krach S, Bedenbender J, Kircher T, Paulus FM, et al Test-retest reliability of fMRI brain activity during memory encoding. Front Psychiatry. 2013;4: 1–9. 24367338 10.3389/fpsyt.2013.00163 PMC3856399

[45] Nadkarni TN, Andreoli MJ, Nair VA, Yin P, Young BM, Kundu B, et al Usage of fMRI for pre-surgical planning in brain tumor and vascular lesion patients: Task and statistical threshold effects on language lateralization. NeuroImage Clin. Elsevier B.V.; 2015;7: 415–423. 10.1016/j.nicl.2014.12.014 25685705 PMC4310930

[46] Benson RR, FitzGerald DB, LeSueur LL, Kennedy DN, Kwong KK, Buchbinder BR, et al Language dominance determined by whole brain functional MRI in patients with brain lesions. Neurology. 1999;52: 789–809. 10.1212/wnl.52.4.798 10078731

[47] Maldjian JA, Laurienti PJ, Driskill L, Burdette JH. Multiple reproducibility indices for evaluation of cognitive functional MR imaging paradigms. Am J Neuroradiol. 2002;23(6): 1030–1037. . 12063237 PMC7976916

[48] Stippich C, Rapps N, Dreyhaupt J, Durst A, Kress B, Nennig E, et al Localizing and lateralizing language in patients with brain tumors: feasibility of routine preoperative functional MR imaging in 81 consecutive patients. Radiology. 2007;243: 828–836. 10.1148/radiol.2433060068 17517936

[49] Stevens MTR, D’Arcy RCN, Stroink, Clarke DB, Beyea SD. Thresholds in fMRI studies: Reliable for single subjects? J Neurosci Methods. Elsevier B.V.; 2013;219: 312–323. 10.1016/j.jneumeth.2013.08.005 23958749

[50] Genovese CR, Noll DC, Eddy WF. Estimating test-retest reliability in functional MR imaging. I: Statistical methodology. Magn Reson Med. 1997;38: 497–507. fmri_Mary M-Converted #362; Used to be #2377. 9339452 10.1002/mrm.1910380319

[51] Liou M, Su H-R, Lee J-D, Aston J a D, Tsai AC, Cheng PE. A method for generating reproducible evidence in fMRI studies. Neuroimage. 2006;29: 383–395. 10.1016/j.neuroimage.2005.08.015 16226893

[52] De Benedictis A, Moritz-Gasser S, Duffau H. Awake mapping optimizes the extent of resection for low-grade gliomas in eloquent areas. Neurosurgery. 2010;66: 1074–1084. 10.1227/01.NEU.0000369514.74284.78 20386138

[53] Hervey-Jumper SL, Li J, Lau D, Molinaro AM, Perry DW, Meng L, et al Awake craniotomy to maximize glioma resection: methods and technical nuances over a 27-year period. J Neurosurg. 2015;123: 325–39. 10.3171/2014.10.JNS141520 25909573

[54] Kim SS, McCutcheon IE, Suki D, Weinberg JS, Sawaya R, Lang FF, et al Awake craniotomy for brain tumors near eloquent cortex: Correlation of intraoperative cortical mapping with neurological outcomes in 309 consecutive patients. Neurosurgery. 2009;64: 836–845. 10.1227/01.NEU.0000342405.80881.81 19404147

[55] Sacko O, Lauwers-Cances V, Brauge D, Sesay M, Brenner A, Roux FE. Awake craniotomy vs surgery under general anesthesia for resection of supratentorial lesions. Neurosurgery. 2011;68: 1192–1198. 10.1227/NEU.0b013e31820c02a3 21273923

[56] Tuominen J, Yrjänä S, Ukkonen A, Koivukangas J. Awake craniotomy may further improve neurological outcome of intraoperative MRI-guided brain tumor surgery. Acta Neurochir (Wien). 2013;155: 1805–1812. 10.1007/s00701-013-1837-3 23955509

